# Effect of Acupuncture on Functional Connectivity of Anterior Cingulate Cortex for Bell's Palsy Patients with Different Clinical Duration

**DOI:** 10.1155/2015/646872

**Published:** 2015-06-16

**Authors:** Hongli Wu, Hongxing Kan, Chuanfu Li, Kyungmo Park, Yifang Zhu, Abdalla Z. Mohamed, Chunsheng Xu, Yuanyuan Wu, Wei Zhang, Jun Yang

**Affiliations:** ^1^Medical Information Engineering, Anhui University of Chinese Medicine, Hefei, Anhui 230012, China; ^2^Laboratory of Digital Medical Imaging, Medical Imaging Center, The First Affiliated Hospital of Anhui University of Chinese Medicine, Hefei, Anhui 230031, China; ^3^Department of Biomedical Engineering, Kyung Hee University, Yongin 446701, Republic of Korea

## Abstract

Acupuncture is widely used in the treatment of Bell's palsy (BP) in many countries, but its underlying physiological mechanism remained controversial. In order to explore the potential mechanism, changes of functional connectivity (FC) of anterior cingulate gyrus (ACC) were investigated. We collected 20 healthy (control group) participants and 28 BP patients with different clinical duration accepted resting state functional MRI (rfMRI) scans before and after acupuncture, respectively. The FC of ACC before and after acupuncture was compared with paired *t*-test and the detailed results are presented in the paper. Our results showed that effects of the acupuncture on FC were closely related to clinical duration in patients with BP, which suggested that brain response to acupuncture was closely connected with the status of brain functional connectivity and implied that acupuncture plays a homeostatic role in the BP treatment.

## 1. Introduction

Bell's palsy is a unilateral idiopathic and mostly transient facial paralysis resulting from dysfunction of the cranial nerve VII (the facial nerve), with a pure peripheral deefferentation [1]. Previous researches have demonstrated that the cortical reorganization may complement the recovery from facial nerve palsy with the change of functional connectivity (FC), mainly demonstrated as disruption at the early stage and enforcement at the later stage [2] in the areas related to error detection, sensorimotor integration, motor integration, and control. The FC [2], defined as the temporal correlation between spatially remote neurophysiological events, has become a significant method for studying neuroplasticity to detect changes during cortical reorganization.

Acupuncture, as an alternative and complementary therapeutic intervention, is playing an increasingly important role in treating BP [3, 4]. However, effectiveness and underlying mechanism of acupuncture for treating BP remain controversial and need further investigation. By now, only a few studies have investigated the effect of acupuncture on resting state functional magnetic resonance imaging (rfMRI) FC of BP patients. Our preliminary research concerning the instant effect of acupuncture in BP treatment [5] reported negative activation in the early stage and positive activation in the later stage of BP. Besides, another investigation [6] of our group showed that changes in FC of the primary somatosensory cortex (SI) induced by acupuncture varied with clinical durations of BP, mainly displayed as decreased connectivity in the early stage but increased connectivity in the later stage. Furthermore, our rfMRI FC studies [7] reported that FC of ACC showed positive correlation with the duration of BP, which suggested that ACC may play a crucial role in the process of cortical reorganization during the recovery from BP. Based on these critical conclusions, in order to probe the effect of acupuncture on the FC of BP patients and further reveal the underlying role of acupuncture in the cortical reorganization in the recovery process of BP, we investigated longitudinal changes of FC (before versus after acupuncture) of bilateral ACC for BP patients with different clinical duration.

The hypothesis of the present research was that acupuncture may have different effect on the FC of ACC for patients with different clinical duration during the recovery of BP.

## 2. Materials and Methods

### 2.1. Subjects

All subjects recruited in this study were right-handed with no histories of drug abuse and no mental, central nervous system or other serious disease. The subjects were divided into two groups: the patient group (28 cases, totally 58 times MRI scanning) and the healthy control group (20 cases, totally 20 times MRI scanning).

**Figure 1:**
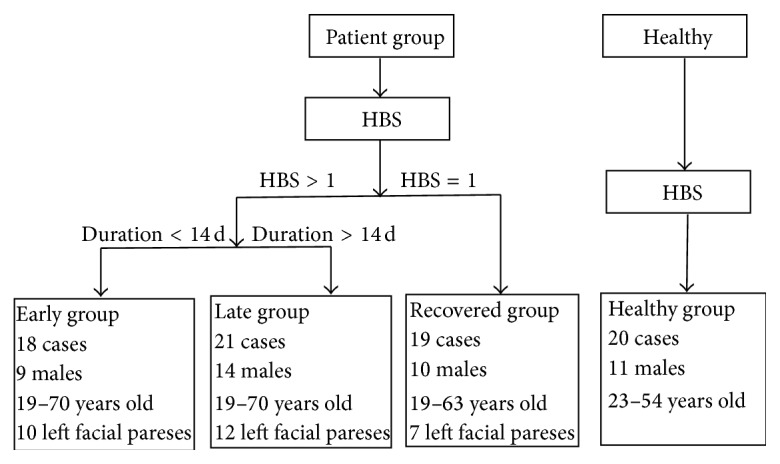
Healthy group and four subgroups of patient group classified based on House-Brackmann score (HBS) and disease duration.

The patient groups, including patients with left and right unilateral facial paresis, were further divided into three subgroups based on the disease duration and HBS scores (House-Brackmann facial nerve grading system, 1 = normal facial movement, and 6 = no movement, scored by an experienced acupuncturist with no prior knowledge about the data results). The 3 subgroups (stages) were the early group (18 cases, 9 males, 19–70 years old; duration < 14 d, HBS > 1; 10 left facial pareses), the late group (21 cases, 14 males, 19–70 years old; duration > 14 d, HBS > 1; 12 left facial pareses), and the recovered group (19 cases, 10 males, 19–63 years old; HBS = 1; 7 left facial pareses). Among the 28 subjects of the patient group with different clinical duration, 4 undertook MRI scanning only once, 18 undertook it twice, and 6 undertook it thrice. Manual acupuncture was applied to all patients thrice a week semi-individually at acupoints chosen by acupuncturists based on the individual symptoms in the course of acupuncture treatment. The acupoint Hegu (LI4) was selected in present experiment for all subjects since LI4 is usually selected as the main acupoints in the clinical treatment of BP. All healthy subjects (20 cases, 11 males, 23–54 years old) were either college students or the workers in the hospital. All subjects signed informed consent forms before participating in the experiment in accordance with the Human Research Committee of the First Affiliated Hospital of Anhui University of Traditional Chinese Medicine (see Figure 1).

### 2.2. fMRI Data Acquisition

The experiment was performed in the MRI room of the Medical Imaging Center, the First Affiliated Hospital of Anhui University of TCM. The Siemens Symphony 1.5 T MRI whole body scanner and standard head coil were used. All subjects were instructed to lie down with eyes closed and to stay awake. All lights in the scanning room were turned off to avoid unwanted visual stimulation.

**Figure 2:**
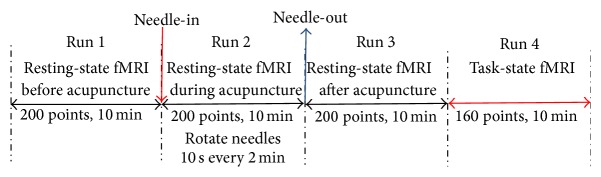
fMRI paradigms of the experiment.

Eight sequences were scanned: (1) pilot images; (2) T2-weighted images to rule out any disease of the brain; (3) T1-weighted 2D anatomical images with the axial position parallel to the AC-PC line; the images include 36 slices that covered the whole brain. T1-weighted spin-echo sequence was used, with TR/TE = 500/12 ms, FOV = 230 mm × 230 mm, slice thickness/interval = 3.0 mm/0.75 mm, and resolution = 192 × 144; (4) resting-state fMRI before acupuncture, namely, Run 1; the EPI BOLD sequence with TR/TE/FA = 3000 ms/30 ms/90°  was used and FOV = 192 mm × 192 mm and resolution = 64 × 64; (5) resting-state fMRI with the same parameters during acupuncture, namely, Run 2; (6) resting-state fMRI with the same parameters after acupuncture, namely, Run 3; (7) task-state acupuncture fMRI: the scanning direction and the number of slices were the same as those of the resting-state fMRI, with TR/TE/FA = 4000 ms/50 ms/90°, namely, Run 4; (8) T1-weighted 3D anatomical images: the sagittal position was taken, and total of 176 slices were scanned which covered the whole brain. The spoiled gradient echo sequence was used, with TR/TE/FA = 2100 mm/3.93 mm/13°, FOV of 250 × 250 mm, slice thickness/spacing = 1.0 mm/0.5 mm and resolution of 256 × 256. It took about 60 minutes to complete all of the data acquisition. fMRI paradigms are shown in Figure 2.

### 2.3. Extraction of the Region of Interest

Bilateral ACC was extracted as region of interest (ROI) for FC analysis. The datasets were from 37 healthy volunteers with the task of mouth movement. The paradigm for motor task lasted 400 seconds, which consists of 40-time task of opening and shutting mouth (mean time 7.8 ± 1.6 s) separated by 2-second duration. The volunteers were trained to open or shut mouth when seeing the word (outward/protrude) shown randomly on the display before scanning and were instructed to lie down on the scanning bed and keep their body static.

The experiment was completed in the Department of Biomedical Engineering of Kyung Hee University of South Korea. The Philips Achieva 3.0 T MRI whole body scanner and 8-channel head coil were used. A total of 3 sequences were scanned, which were (1) 2D structural image: TR/TE of 2000 ms/35 ms, voxel size of 2.785 mm × 2.875 mm × 4 mm, slice/volume of 34/180, and matrix 80 × 80; (2) EPI-BOLD: TR/TE of 2000 ms/35 ms, voxel size of 2.785 mm × 2.875 mm × 4 mm, slice/volume of 34/200, and matrix of 80 × 80; (3) T1-Weighted 3D structural image: TR/TE of 2000 ms/35 ms, slice/gap of 1.0 mm/1.0 mm, and matrix of 256 × 256.

To investigate the changed FC of bilateral ACC with related brain areas, the ROIs were extracted from the statistic activation maps obtained from motor task experiment. The maximum point of activation strength of bilateral ACC, along with its 33 neighbors, was selected as ROIs (a sphere with radius as 4 mm and voxel size 2 × 2 × 2 mm), as shown in Figure 1 of [7].

### 2.4. Paradigms of the Experiment

The acupuncture in the experiment was executed by a professional acupuncturist. Resting-state fMRI data before acupuncture (Run 1) lasts for 10 min (200 points). Then, the needle was inserted into the acupoints of LI4 on the contralateral hand of the paralyzed face and rotated to achieve De-Qi sensation. The second fMRI data (Run 2) was collected including 200 points for 10 min, during which the needle was rotated bidirectionally for 10 s every 2 min. Then the needle was pulled out and the third fMRI data after acupuncture (Run 3) including 200 points was obtained.

### 2.5. Data Preprocessing

Data analysis was performed using the FSL (Oxford Centre for Functional MRI of the Brain's (FMRIB's) Software Library), Freesurfer, and AFNI. The preprocessing was applied as follows. Anatomical images were reconstructed using Freesurfer recon-all and then tilt correction was done for functional and anatomical images using 3drefit and fslorient. Nonbrain removal was done using mri_watershed for anatomical images and BET for functional images. Motion correction was done using MCFLIRT (Motion Correction using FMRIB's Linear Image Regression Tool) and melodic to compensate for any head movements during the scan. The functional images were then coregistered to the high-resolution anatomical images reconstructed by Freesurfer. Afterwards, the functional images were registrated to standard MNI152 space (Montreal Neurological Institute) using FNIRT and FLIRT (affine transformation with FMRIB's Linear Image Registration Tool). Functional data were smoothed using a Gaussian kernel of FWHM 6 mm; and band-pass filter (0.007 HZ < *f* < 0.1 HZ) was also performed to reduce the effect of low-frequency drift and high-frequency noise. Then, individual data of right-sided facial palsy patients were flipped along the *y*-axis so that all data could be processed unilaterally. The individual 4D data was then used for further group statistics and connectivity analysis.

### 2.6. Functional Connectivity Analysis

In our research, to compute the FC of ACC, the temporal signal series of cerebrospinal fluid, white matter was extracted firstly. Then, based on linear regression analysis, variances including 6 parameters obtained by rigid body correction of head motion and the signal of cerebral spinal fluid and white matter were removed. Finally, individual statistical maps were obtained based on the general linear model for further group analysis.

### 2.7. Group Analysis

Standard space parameters of individual subjects were imported to a high level analysis with FLAME (FMRIB's Local Analysis of Mixed Effects). With the aim of investigating the effects of acupuncture on FC of BP patients with different clinical durations, we conducted intergroup analysis through paired *t*-test before and after acupuncture for each group. The individual data with head movement more than 2 mm or 2° were excluded before group analysis. The significance threshold for FEAT was set at *z* = 2.3 and *P* = 0.01. All results were then corrected using cluster (based on theory of Gaussian Random Field, GRF) to obtain the activation maps. The results of intergroup analysis were corrected using Monte-Carlo simulations with *P* = 0.01, *α* = 0.05, and cluster size = 68.

## 3. Results

There were no significant difference among subjects' age distributing and sample size of four groups. To address the significant differences in functional connectivity changes among different groups, the results of group analysis for each group were showed as follows.

### 3.1. The Healthy Control Group

Paired *t*-test was done before and after acupuncture to find out significant changes induced by acupuncture effect on FC of bilateral ACC of the healthy group. In the healthy group, after being corrected with Monte-Carlo method (*P* = 0.01, *α* = 0.05 and cluster size = 68), nostatistical significant differences were observed for bilateral ACC before and after acupuncture. The results imply that acupuncture has no significant effect on FC of bilateral ACC in healthy subjects.

### 3.2. The Early Group

**Figure 3:**
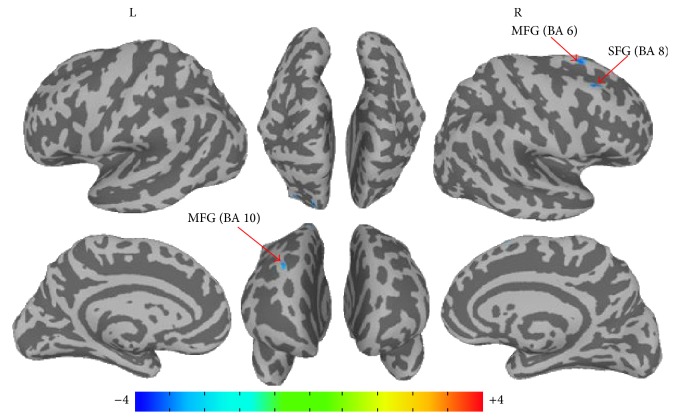
Changed functional connectivity of right ACC after acupuncture for patients with Bell's palsy at early stage. *P* ≤ 0.01, *α* ≤ 0.05, corrected with Monte-Carlo method. BA: Brodmann area; SFG: superior frontal gyrus; MFG: right middle frontal gyrus; MFG: middle frontal gyrus.

Different results were observed for the early group. In the early group, after being corrected with Monte-Carlo method (*P* = 0.01, *α* = 0.05, and cluster size = 68), significant changed FC were found after acupuncture. For the left ACC (ipsilateral to Bell's palsy), no remarkable changes of FC were found. Significant decreased connectivity of the right ACC (contralateral to BP) after acupuncture were observed in right superior frontal gyrus (SFG, BA 8), right middle frontal gyrus (MFG, BA 6), and right middle frontal gyrus (MFG, BA 10) (as shown in Figure 3 and Table 1).

### 3.3. The Latter Group

**Figure 4:**
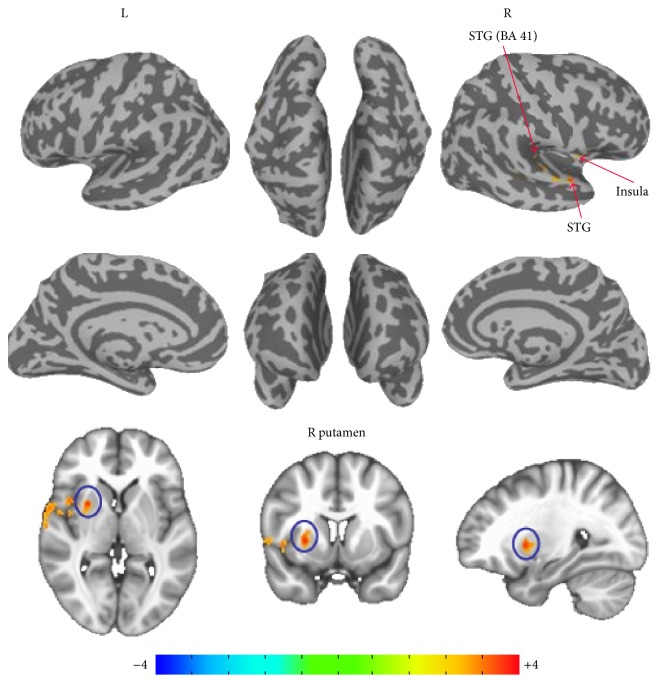
Changed functional connectivity of right ACC after acupuncture for patients with Bell's palsy at later stage. *P* ≤ 0.01, *α* ≤ 0.05, corrected with Monte-Carlo method. BA: Brodmann area; STG: superior temporal gyrus; STG: superior temporal gyrus.

For the latter group, after being corrected with Monte-Carlo method (*P* = 0.01, *α* = 0.05, and cluster size = 68), FC of ACC was also changed after acupuncture. For the left ACC (ipsilateral to Bell's palsy), no significant changes of FC were found. Significant increased FC of the right ACC were observed in right superior temporal gyrus (STG, BA 22), right insula (BA 22), right superior temporal gyrus (STG, BA 41), and right putamen (see Figure 4 and Table 2).

### 3.4. The Recovered Group

Similar with the healthy group, in the recovered group, the intergroup analysis results before and after acupuncture with Monte-Carlo correction (*P* = 0.01, = 0.05, and cluster size = 68) showed no significant functional connectivity changes of bilateral ACC.

## 4. Discussions

In this study, in order to assess the role of acupuncture during the recovery of BP, we investigated the FC changes of ACC induced by acupuncture for patients with different clinical duration. The results suggest that the acupuncture effects on the FC varied with clinical duration.

### 4.1. Overall Acupuncture Effect on ACC Connectivity in Bell's Palsy Patients with Different Clinical Durations

As well known, one of the most important characteristics of BP is the damaged efferent nerve (without afferent nerve) and the consequent impaired facial motor function of the affected side of the face. Therefore, the sensory feedback of the acute reduction of facial motor performance due to BP will be detected by brain, thus causing increased processing in brain areas responsible for the monitoring and integration of somatosensory and motor information. Previous functional neuroimaging studies have shown that the ACC is important in error detection and performance-monitoring functions, including executive function, response selection, and conflict monitoring [8].

The present results indicated that no changes were found for the FC status of bilateral ACC for both the healthy and the recovered groups after acupuncture. Presently, researches show that the therapeutic principles of acupuncture are not through relieving local condition of the diseased area but in the way of reestablishing the balance of the internal milieu (involving Ying/Yang, the Five Elements, and the Zhong-Fus) [9]. Therefore, for healthy subjects and the recovered group, their homeostatic can be considered to stay in/in return to a balance state; thus no significant effect on FC of ACC was observed after acupuncture.

Acupuncture-induced FC changes of the contralateral ACC were observed for both the early and the later groups. As we know, one of the most remarkable features of the human brain is its ability to adapt to new situations and to new information. Changes in the functional network connectivity status, which reflect the process of cortical reorganization of different brain areas, may give expression to this feature. It is suggested that [1] the functional reorganization caused by transient peripheral deefferentation, which can be interpreted as the compensatory effect of brain to the impaired motor performance of BP patients, happened after Bell's palsy. As a result of this process, the connectivity status of the brain changed and thus led to a different acupuncture response of brain. Generally speaking, compared with the healthy group, FC changes in the early and the late group imply the different acupuncture responses resulting from the changed FC status of BP patients. The results here are consistent with our previous research concerning the instant effect of acupuncture in BP treatment, which reported the conclusion that the brain responses to acupuncture differ at different pathological statuses and probably depend on the brain functional status [1].

Another remarkable characteristic of our results is that the acupuncture-induced changes of the intrahemispheric FC were found only for the contralateral ACC but not for the ipsilateral ACC for all groups. Previous investigations have consistently reported that the changed brain responses to acupuncture were mainly contralateral to the palsy area [2]. An fMRI study on BP [2] also revealed a significant acutely disrupted but unaltered interhemispheric connectivity of MI and other parts of the facial motor network at early stage of the palsy, followed by a return toward normal during the course of recovery. Their results are in accordance with ours. This can be explained since motor commands of paretic side are blocked in the acute stage of BP; then the error in the process of command execution was feed backed to the contralateral ACC and thus the FC status changed due to various adjustment and compensatory mechanism. No changes of the FC status of the ipsilateral ACC may indicate that the brain areas related to the healthy side remain in a good condition.

### 4.2. Changed Functional Connectivity of Sensorimotor Related Brain Areas

Compared with the Pre-Acu, decreased FC was found in sensorimotor related brain areas including SFG (BA 8) and MFG (BA 6, BA 10) after acupuncture. BA 8 and BA 6 are well known as the advanced motor center in planning, integration, and execution of motor function. The difficulty in movement of the paralyzed facial muscle in patients with BP may elicit FC changes of these motor related areas. Studies concerning the BP suggested that the impaired motor function (without a lesion in the brain) might initially lead to a disrupted connectivity within the cortical facial motor network, with a subsequent reorganization supporting functional recovery [1, 2]. Our research concerning acupuncture-induced FC changes of SI also reported decreased connectivity in the early stage and increased connectivity in the later stage [1]. Therefore, hypoactivation of motor related areas after acupuncture may result from disruption FC in the early stage of Bell's palsy.

Another sensorimotor correlated brain area with changed FC was observed in putamen at the latter group. While it has been concluded that the putamen has no specific specialization, it works with many other motor related structures to control many types of motor skills, including motor learning, motor performance and tasks [10], and motor preparation [11]. In addition, the putamen contains high levels of opioid receptors and is considered to be involved in sensory and emotional components of pain. Meanwhile, fMRI research of patients with PD received acupuncture treatment demonstrated that the activated putamen was correlated with enhanced motor function of patients. Based on comprehensive analysis, increased FC in putamen may result from the cortical reorganization in motor correlated brain areas which were caused by the loss of facial motor control in BP patients.

In addition, increased FC was observed in superior temporal gurus (STG, BA 22, and BA 41), which is well known as the primary auditory cortex and is involved in auditory and language processing. Changed FC in temporal lobe reflects brain's potential compensatory mechanism because of facial motor difficulty in BP patients. Besides, changed FC in STG (BA 22) may also occur as cortical reorganization result from the difficulty in flexible pronunciation of those patients with serious facial paralysis.

### 4.3. Changed Functional Connectivity of Homeostatic Related Network

Changed connectivity between the contralateral ACC and insular cortex induced by acupuncture is also an interesting finding in our research. As well known, the insular cortex is involved in a wide range of functions including motor control and homeostatic regulation [12–15]. As reported in previous neuroimaging researches, the ACC and insular cortex play an important role in the network termed as the “homeostatic afferent processing network” [16]. The network represents all aspects of the physiological condition of the body and meanwhile provides crucial sensory input that is essential for maintaining homeostasis. The fMRI research in functional diarrhea reported that acupuncture brings functional connectivity changes to the homeostatic afferent processing network only in patients but not in the healthy group because of functional abnormality of the network in functional diarrhea patients. The results were in accordance with ours since the changed FC induced by acupuncture within the homeostatic afferent processing network in BP patients may suggest the unbalanced functional state result from the Bell's palsy. All results imply the homeostatic regulation role of acupuncture that is emphasized in the traditional Chinese medical theory. But considering the high self-recovery rate of Bell's palsy and there was no control group in our experiment, the results might just reflect the self-recovery of the disorder instead of the acupuncture effect.

## 5. Conclusion and Limitation

The paper introduces the influence of acupuncture on FC of ACC for BP patients with different clinical duration and our results indicate that different changes were observed. Changed FC induced by acupuncture occurred in more than one functional neural system. The deactivation in the early group and positive activation in the latter group kept peace with the disrupted connectivity in the acute stage and subsequent reorganization during the recovery of the BP. This synchronization suggested the dependence of acupuncture effect on the functional status of brain and may imply the homeostatic role of acupuncture in BP treatment. Moreover, changed FC of “homeostatic afferent processing network” induced by acupuncture also reflected homeostatic regulation role of acupuncture.

Present results indicate that acupuncture might at least partly contribute to the functional recovery of BP arising from the cortical reorganization by changing the FC within the somatosensory motor network and other related areas. The evidence provided in this research for the effectiveness of acupuncture in treating BP is limited because there was no blank control group due to ethics restriction. Therefore, we cannot exclude the possibility that the results might just reflect the self-recovery of Bell's palsy. The paper may bring to light the underlying mechanism of acupuncture during the recovery of BP, although further researches are still needed.

## Figures and Tables

**Table 1:** Group analysis of areas changed FC with right ACC after acupuncture for patient with Bell's palsy in the early group.

Region (BA)	Side	BA	Coordinate (MNI)			*Z* value	Cluster size
			Peak *x* (mm)	Peak *y* (mm)	Peak *z* (mm)		
Superior frontal gyrus	R	8	32	28	56	−4.151	143
Middle frontal gyrus	R	6	26	18	62	−3.643	126
Middle frontal gyrus	R	10	38	30	46	−3.515	77

BA: Brodmann area; L: left; R: right; the threshold was set at *P*≦0.01; *α*≦0.05 (corrected with Monte-Carlo method).

**Table 2:** Group analysis of areas changed FC with right ACC after acupuncture for patient with Bell's palsy in the latter group.

Region (BA)	Side	BA	Coordinate (MNI)			*Z* value	Cluster size
			Peak *x* (mm)	Peak *y* (mm)	Peak *z* (mm)		
Superior temporal gyrus	R	22	58	2	−4	3.945	130
Insula	R	22	46	6	−4	3.633	102
Superior temporal gyrus	R	41	46	−34	12	3.806	88
Putamen	R		28	8	2	3.626	68

BA: Brodmann area; L: left; R: right; the threshold was set at *P*≦0.01; *α*≦0.05 (corrected with Monte-Carlo method).
